# Egas Moniz: 90 Years (1927–2017) from Cerebral Angiography

**DOI:** 10.3389/fnana.2017.00081

**Published:** 2017-09-19

**Authors:** Marco Artico, Marialuisa Spoletini, Lorenzo Fumagalli, Francesca Biagioni, Larisa Ryskalin, Francesco Fornai, Maurizio Salvati, Alessandro Frati, Francesco Saverio Pastore, Samanta Taurone

**Affiliations:** ^1^Department of Sensory Organs, Sapienza University of Rome Rome, Italy; ^2^Department of Anatomy, Histology, Forensic Medicine and Orthopedics, Sapienza University of Rome Rome, Italy; ^3^IRCCS Neuromed Pozzilli, Italy; ^4^Department of Translational Research and New Technologies in Medicine and Surgery, University of Pisa Pisa, Italy; ^5^Department of Systems Medicine, University of Rome Tor Vergata Rome, Italy

**Keywords:** angiography, history of anatomy, imaging methodology, neuroimaging, neurosurgery, neuroanatomy

## Abstract

In June 2017 we celebrate the 90th anniversary of the pioneer discovery of cerebral angiography, the seminal imaging technique used for visualizing cerebral blood vessels and vascular alterations as well as other intracranial disorders. Egas Moniz (1874–1955) was the first to describe the use of this revolutionary technique which, until 1975 (when computed tomography, CT, scan was introduced in the clinical practice), was the sole diagnostic tool to provide an imaging of cerebral vessels and therefore alterations due to intracranial pathology. Moniz introduced in the clinical practice this fundamental and important diagnostic tool. The present contribution wishes to pay a tribute to the Portuguese neurosurgeon, who was also a distinguished neurologist and statesman. Despite his tremendous contribution in modern brain imaging, Egas Moniz was awarded the Nobel Prize in Physiology or Medicine in 1949 for prefrontal leucotomy, the neurosurgical intervention nowadays unacceptable, but should rather be remembered for his key contribution to modern brain imaging.

## Introduction

The year 2017 marks the 90th anniversary of the pioneer discovery of cerebral angiography, the original imaging technique used for visualizing cerebral blood vessels and their alterations as well as intracranial pathologies which alter the course and/or ramifications or caliber of blood vessels in the brain.

Antonio Caetano de Abreu Freire Egas Moniz (1874–1955) was the first to describe the use of such a revolutionary imaging technique. Until 1975, when computed tomography (CT) scan was introduced into clinical practice, cerebral angiography was the sole diagnostic technique to provide a reliable investigation of intracranial disorders. The present contribution is meant as a tribute and an expression of admiration for the Portuguese neurosurgeon, neurologist and statesman who introduced this fundamental tool into clinical practice. Cerebral angiography is still a seminal diagnostic tool whose sensitivity is increased by technological advancements.

### The Early Career in Medicine and the Intense Political Commitment

**Figure 1:**
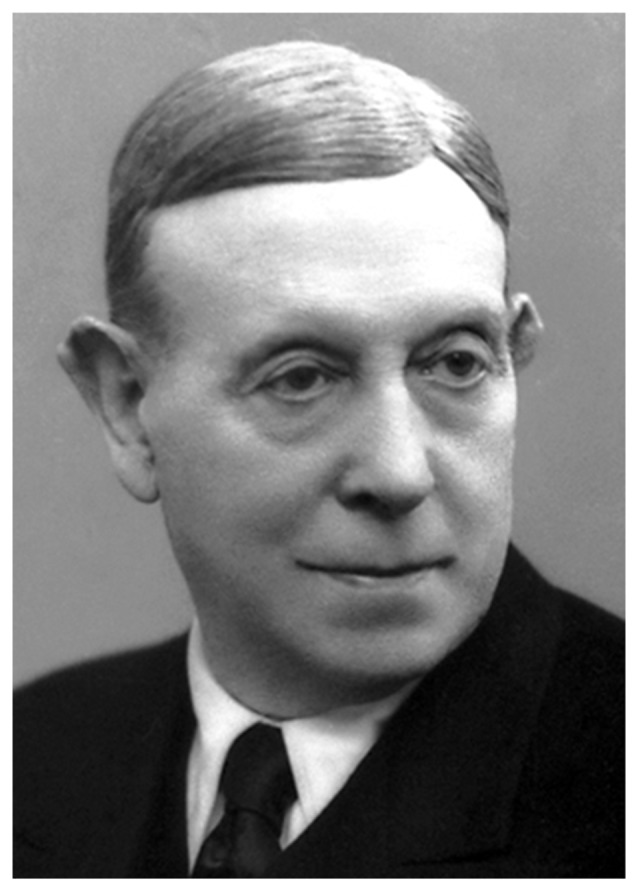
A picture of Professor Antonio Caetano de Abreu Freire Egas Moniz. Kindly provided by the Library of the School of Medicine, University of Pisa.

Egas Moniz (Figure 1), the first Portuguese citizen to be awarded the Nobel Prize in Medicine, was born on November 29th, 1874 in the village of Avança, in northern Portugal. Here, he grew up in the family farm with his parents, Fernando de Pina Rezende Abreu and Maria do Rosario de Almeida e Sousa, and his uncle, Abbé Caetano de Pina Rezende Abreu Sa Freire, who gave him early education, before the young Egas Moniz was admitted to the University of Coimbra. After his University studies, he signed all his writings with the name of Egas Moniz, a name his godfather gave him to honor the 12th-century Portuguese heroic nobleman who played a major role in the Portuguese resistance against the Moors (Tondreu, 1985).

When he entered the University of Coimbra at the age of 17, Moniz studied mathematics, considering a possible career in Engineering, but he soon decided to join the Faculty of Medicine. In 1899, at the age of 25, Moniz received his MD degree, with honors. After choosing neurology as his specialty, Moniz traveled to France and went to Paris and Bordeaux, where he further trained with renowned French doctors. Indeed, his goal was to improve his knowledge by working with leading figures in neurology and psychiatry: Joseph F. F. Babinski (1857–1932), Joseph J. Dejerine (1849–1917), Pierre Marie (1853–1940) and Jean-Athanase Sicard (1872–1929). Then, Moniz returned to Portugal and in 1902 became Professor at the University of Coimbra. In the same year, he married Elvira de Macedo Dias. In 1911, he was appointed to the Chair of the newly instituted department of Neurology at the University of Lisbon, an academic position he hold until his retirement, in 1945.

During his youth Moniz, who was clearly a gifted with a brilliant intellect and an undisputable talent, developed various interests and was deeply involved in the political life of his country. For several years he took an active part in politics. During certain periods of his life, political activities even prevailed over medical studies (Gorelick and Biller, 1991). Since he was a young medical student, Moniz was a democrat activist and he fought against the Portuguese monarchy. His strong temperament led him to hold tenaciously his political positions up to the extreme consequences; for instance he was imprisoned three times during the student protests at the Medical School of the University of Lisbon (Haas, 2003). Between 1903 and 1917 he served in the Portuguese Chamber of Deputies several times. He established the “Partido Republicano Centrista” (Center Republican Party), and he represented the Republican Party in the Portuguese parliament from 1903 to 1917. He was also appointed as Portuguese Ambassador in Spain in 1917–1918. In 1917, Moniz was also appointed as Minister for Foreign Affairs. In particular, he led the Portuguese delegation at the Paris peace conference held in 1918, at the end of World War I, being Portugal’s signatory to the Treaty of Versailles. In 1919, after a duel caused by a political quarrel, Moniz retired from politics. After the establishment of the dictatorial regime of António de Oliveira Salazar following a military coup d’état He left the political scene, disenchanted and extremely disappointed. At this time, at the age of 52, Moniz devoted himself entirely to research. During his early research activity at the University of Lisbon, he was fascinated by neurological research and he suggested to use X-rays as a method of making brain vessels visible and to localize cerebral neoplasms (Jefferson, 1955). His intuition is reported in his article of 1927: “*Nous avons pris une nouvelle route dans l’espoir d’obtenir la visibilité du cerveau par l’opacité de ses vaisseaux et sourtout de ses artères*. […] *Nous avons pensé que, si nous réussissions à montrer le réseau artériel cérébral, on pourrait aussi faire la localization des tumeurs par les altérations qu’elle montreraient dans la contexture de la charpente artérielle*”. (Moniz, 1927a).

### Visualizing the Cerebral Vessels: The “Arterial Encephalography”

**Figure 2:**
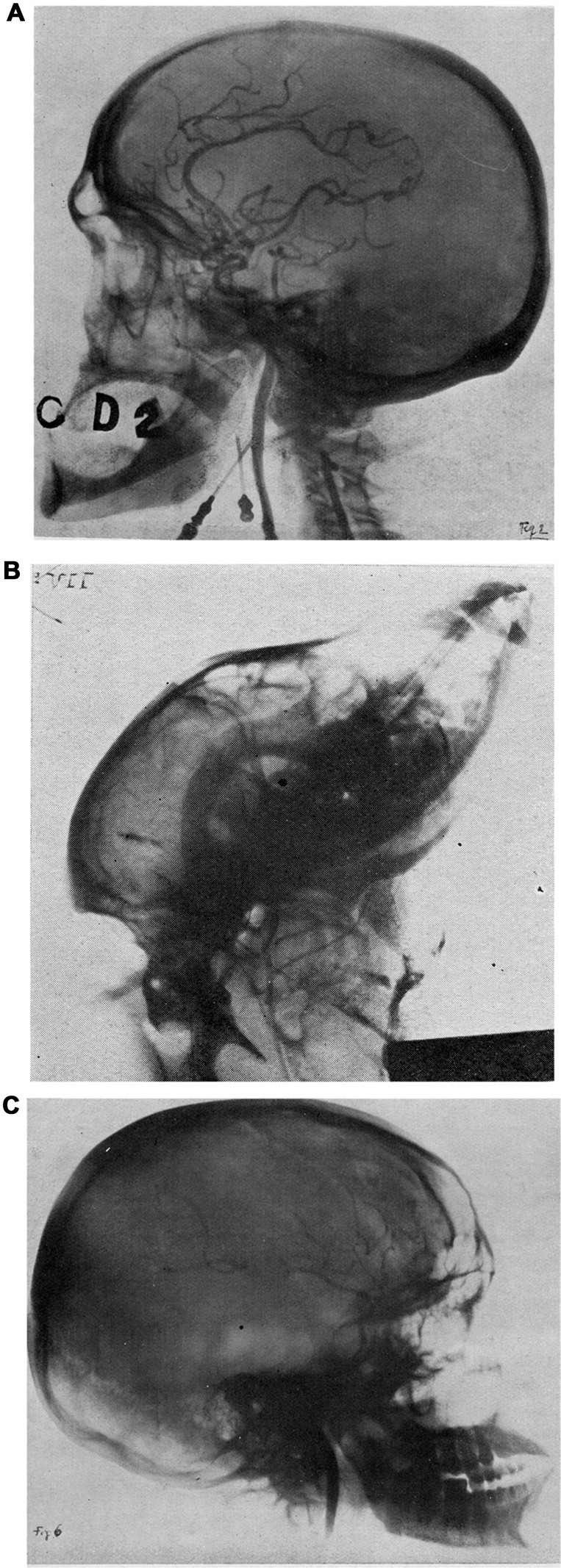
**(A)** The arterial network arising from the internal carotid artery, as visualized following the injection of 30% sodium iodide (NaI) in a formalin-fixed head (from “*L’encéphalographie artérielle, son importance dans la localization des tumeurs cérébrales*”, Egas Moniz (de Lisbon), Société de Neurologié, séance du 7 Juillet 1927; Revue Neurologique, T. II, Figure 2, n° 1 Juillet, 1927). **(B)** The visualization of some cerebral arteries in a living dog (from “*L’encéphalographie artérielle, son importance dans la localization des tumeurs cérébrales*”, Egas Moniz (de Lisbon), Société de Neurologié, séance du 7 Juillet 1927; Revue Neurologique, T. II, Figure 5, n° 1 Juillet, 1927). **(C)** First successful “arterial encephalography” in a 20 year-old man with a pituitary tumor and a Babinski-Frohlich’s syndrome (from “*L’encéphalographie artérielle, son importance dans la localization des tumeurs cérébrales*”, Egas Moniz (de Lisbon), Société de Neurologié, séance du 7 juillet 1927; Revue Neurologique, T. II, Figure 6, n° 1 Juillet, 1927). Kindly provided by the Library of the School of Medicine, University of Pisa.

During his first years of residency in Neurology, he went to France, where he could train with renowned doctors in the main neurological centers in Paris and Bordeaux. During this period he met Jean-Athanase Sicard, Professor of the Faculty of Medicine in Paris, who first introduced the use of lipiodol for the diagnosis of medullary compression (Sicard and Forestier, 1921). Moniz was so impressed by Sicard’s technique that he started thinking about the possibility to apply a similar technical approach to the visualization and localization of brain tumors. When Moniz approached neurology, the diagnostic technique used to locate intracranial tumors was only based on pneumo-encephalography. This method developed by the American neurosurgeon Dandy (1918) consists in the injection of air into the brain ventricles. In order to improve brain imaging, Moniz took advantage of the previous studies carried out by Sicard and Forestier (1921, 1925, 1928) and they laid the basis for the creative logical intuition that even brain blood vessels may be imaged by injecting specific substances being opaque to X-rays. Moniz named this procedure as “*encéphalographie artérielle*” (arterial encephalography), a term which was meant to emphasize the sharp imaging of blood vessels instead of air, as in pneumo-encephalography (Moniz, 1927a, 1931, 1934; Lima, 1973; Duarte and Goulão, 1997). Then, during the early 1920s, Moniz focused his research on the best suitable radio-opaque solution to be injected into the arteries. As he wrote in his article published in Revue Neurologique of 1927, the dye had to meet some specific technical requirements: “*une substance opaque, non huileuse, qui pourrait facilement passer par les capillaires, de façon à éviter toute espèce d’embolie et inoffensive*” (Moniz, 1927a). He thus experimented various dyes in order to enhance the amount of contrast after X-rays penetration and, therefore, to improve the visualization of brain vessels and brain blood vs. brain tissue (Moniz, 1927a,b). At first, he selected lithium bromide, strontium bromide and sodium iodide and he performed different experiments, both in cadavers, animals (i.e., rabbits and, particularly, dogs) and humans (Figure 2). The aim was to determine the toxicity of each contrast solution as well as the percentage of various radio-opaque compounds within these solutions. This was critical considering that any given concentration prepared at the bench was further diluted in the blood. During his early attempts, Moniz observed that the intravenous injections of lithium bromide were irritant and caused, albeit transiently, pain in the injected patients. Moreover, intravenous injections of strontium bromide caused local warmth, which only occurred due to rapid administrations of the contrast medium. Then, thanks to the help of his assistant Almeida Lima, he tested the outcomes of intravenous injection of sodium iodide (25% solution) in patients (Lima, 1973; Krayenbühl, 1977). This experiment met noticeable success since the sodium iodide injections were not painful, while being very rapid, and producing an optimal contrast enhancement.

His studies aimed at determining the gold standard radio-opaque injection into arteries. His studies represent a remarkable example of neuroanatomy teaching, while advancing the progress in neuroscience. During these studies Moniz was able to translate a logical algorithm based on basic knowledge of the brain into clinical practice. This process was time consuming and frustrating at the beginning. When he performed the first series of trials in four patients he encountered a failure. He first attempted percutaneous puncture of the internal carotid artery which produced two non-diagnostic (non-contrasted) films and only one film which was fairly contrasted and barely detectable. In one case, Moniz had even to stop the procedure due to blood vessel rupture and spread of the contrast medium in the surrounding tissue. As he concluded in his article, the failure of these experiments was probably due to the use of very thin needles (0.5–0.6 mm of diameter, 5 cm of length), which may have moved out from the vessel during the procedure (Moniz, 1927a). In the second series of trials the internal carotid artery was exposed surgically, and it was temporarily and partially occluded in the proximal segment. Unfortunately, the first patient died 8 h after the procedure due to a stroke. In this case, the artery was injected directly with sodium bromide making it clear that the contrast medium was too dense to allow a physiological blood flow. Therefore, in the last four patients, Moniz changed the contrast medium into sodium iodide (Figure 2). He concluded his article detailing each step of his protocol: “*Pour le moment, la technique que nous conseillons, inoffensive pour les malades et capable de donner une assez bonne encéphalographie artérielle, est la suivant*:

1. *Préparer le malade avec une ou deux injections de morphine et d’atropine*;
2. *Mettre à découvert la carotide interne*;
3. *Fixer la tête du malade sur le châssis photographique par un bandage, pour éviter le déplacement de la tête*;
4. *Faire la piqûre de la carotide sans laisser rentrer le sang dans la seringue*;
5. *Avoir toujour un grand soin pour éviter l’entrée de l’air*;
6. *Pratiquer tout de suite, par une pince, la ligature provisoire de la carotide interne*;
7. *Injecter immédiatement et rapidement 5 à 6 cc. d’une solution d’iodure de sodium (à 25%) récemment préparée et stérilisée*;
8. *Tirer un ou plusieurs instantenés radiographiques (les plus rapidement possible) en continuant à injecter le liquide opaque*;
9. *Défaire tout de suite la ligature temporaire de la carotide interne*” (Moniz, 1927a).

#### The Final Improvement of Cerebral Angiography

After the first series of experiments carried out by injecting bromides into the carotid and vertebral arteries of corpses (Moniz, 1927a; Doby, 1992), the scarce autopsy material led Moniz to perform the technique, directly, on live patients in collaboration with Almeida Lima. The first eight attempts were failures, nonetheless he continued to improve the technical approach with admirable consistency and adamant devotion which led him to success, which was achieved on the ninth trial (Figure 2). Indeed, at the latter attempt, the vascular branches appeared clearly visible and defined on films, and this allowed Moniz to describe, in a very detailed manner, the various arterial displacement caused by the presence of a tumor (Moniz, 1927a). At that time, Moniz was able to decipher details of abnormal vascularization, as occurring in specific brain lesions, such as various kinds of brain neoplasms or aneurysms. He concluded his article as follows: “*La démonstration de notre thèse est faite. On peut obtenir, chez le vivant, la radiartéréographie du cerveau et elle peut nous fournir des éléments pour la localization des tumeurs*.” (Moniz, 1927a). As reported by Wolpert (1999), Moniz was truly a pioneer in cerebral angiography: “after establishing the normal appearances of the carotid arteries in cadavers, and establishing an optimal concentration of a radio-opaque dye to visualize the arteries, Moniz began to perform arterial encephalography in live patients” (Figure 2).

On June 28th 1927 Moniz was finally able to achieve the visualization of a pituitary tumor in a young man (Moniz, 1927a; Figure 2C). On July 7th 1927 his revolutionary discovery was presented at the congress of the Neurological Society in Paris (Moniz, 1927a; Novak, 1982; Bertolote, 2015), thus arousing the interest of famous neurologists such as Babinski, Sicard and Clovis Vincent. After describing the normal distribution of intracranial blood vessels, Moniz obtained images indirectly showing through the use of X-rays the location and size of intracranial tumors. This was initially achieved by measuring the displacement of injected arteries produced by the neoplasm. Moreover, Moniz et al. (1933) further developed an open indirect method for performing vertebro-basilar arteriography, by injecting the contrast into an exposed subclavian artery (Antunes, 1974; Gorelick and Biller, 1991).

### The Nobel Prize in 1949: The Neurologist Awarded as a Psychiatrist

All his activities witnessed the passion of a talented human being for the process of learning and discovery who was in constant search for improvement as a lifestyle and inner need. Over 200 articles regarding normal and pathological cerebral angiography were published by Moniz et al. (1933). His technique was further improved by German and Swedish physicians and was proven to be of undisputable value for the diagnosis of several intracranial disorders. Although he received for three times the nomination for the Nobel Prize (1928, 1932 and 1937) for the discovery of cerebral angiography, he was never awarded for the discovery of this technique (Ligon, 1998; Lass et al., 2012). Despite the importance of his revolutionary diagnostic tool, Moniz won the Nobel Prize for Medicine on 1949 (together with Walter Rudolf Hess) for another technique he had described later: the prefrontal leucotomy (“the white cut”), undoubtedly less important and noteworthy than brain angiography, which was used for the treatment of psychoses (Moniz, 1936a,b; Damásio, 1975; Tierney, 2000; Tan and Yip, 2014). This is an obvious discrepancy if one considers the comparison between a questionable method (prefrontal leucotomy) and a still fundamental and useful technique such as cerebral angiography. In our opinion, there is no doubt whatsoever that the revolutionary contribution and outstanding diagnostic impact of cerebral angiography would certainly have deserved the Nobel Prize. On the other hand, it should be remembered that at that time only a few drugs were available to relieve human psychoses slightly, which may explain why the impact of leucotomy on the scientific community was highly relevant. Probably, as Wolpert reported in his article (Wolpert, 1999), we have to consider that: “if Moniz would attempt to make his experiments today, ethical considerations and restrictions imposed by human investigative review committees would have prevented him from making his major contribution in medical science”. As recently discussed by Csoka (2016), nowadays we are immersed within a continuous stream of data which are supposed to push the progress of medicine ever forward. Nonetheless, some research data flourish while others flounder, which poses the question on what is the distinguishing feature between success and failure within this context. Timing is key and without a delicate analysis of the historical context where the discovery was judged. It is non-cautious to provide a statement of the merits for a Nobel Prize. Thus, it remains difficult to judge and to compare who really deserve a Nobel Prize within a certain time frame. Similarly, the impact of leucotomy is critical to be judged from a non-historical perspective.

This multifaceted perspective is analyzed by Lass et al. (2012) in a article titled: “Egas Moniz: a genius, unlucky looser or a Nobel Committee error?”. This also explains the beauty of a article by Gross and Schäfer (2011) which was dedicated to reconstruct the historical background of Moniz work and Nobel Prize. In the present manuscript we wish to comment how sterile can be to express an opinion on the historical significance of leucotomy while avoiding to understand the grandness of a scientist. Egas Moniz definitely improved the progress in neuroanatomy and neurology from several aspects which surpass the historical context. This is why by celebrating the pioneer discovery of cerebral angiography we wish to honor Egas Moniz brilliant intellect and contribution to mankind beyond any narrow historical background.

Over the past years, his portrait and his pioneering work on angiography have been depicted on several celebratory medical paintings, statues and even on a Portuguese banknote of 10,000 “escudos” issued in 1989 by the “Banco de Portugal” (Bank of Portugal). Furthermore, several Portugal stamps commemorated the first cerebral arteriogram, performed by Moniz (1927a).

## Conclusion

Moniz was an authoritative physician, a remarkable historian, a literary and artistic critic, a brilliant politician and diplomat and a wonderful example of whole intellectual dedication. He was a charismatic person and a gentleman who enjoyed entertaining his guests in his own library, gifted with a rich collection of antiques. He was also writer of several works, including an autobiography. He became paraplegic at the age of 65 after being shot in his office by one of his mentally disturbed, schizophrenic, patients. After this accident, which almost killed him, Moniz was confined in a wheelchair, but he still continued his private medical practice until his death, just when leucotomy was falling into disrepute. On December 13th, 1955, he died peacefully at the age of 82, in the family farm where he was born. The appreciation for his work clearly emerges from the obituary of the British neurologist Sir Geoffrey Jefferson. In The Lancet, Jefferson wrote: “His life was an unusually productive one, his name will live for his two great contributions to medicine […] Humanity has reason to pay its last respects and express its gratitude to another great Portuguese explorer” (Jefferson, 1955). However, even when focusing on a single facet of his multiple activities, we remain in debt for his unquestionable merits in fostering neuroimaging. He was the first to allow anatomists, neurosurgeons and neuroradiologists to “see the brain vessels within the skull”. The chance he provided was unique. Since being developed in 1927, cerebral angiography remained for almost 50 years the main diagnostic tool for detecting intracranial pathology until the introduction of CT in 1975. However, for specific and frequent conditions, brain angiography still has a diagnostic and therapeutic application, which remains unsurpassed.

## Author Contributions

All authors shared important intellectual content and contributed to the historical search and they wish to celebrate the invaluable discovery of Egas Moniz and cerebral angiography.

## Conflict of Interest Statement

The authors declare that the research was conducted in the absence of any commercial or financial relationships that could be construed as a potential conflict of interest.
